# Magnetic resonance spectroscopy as a prognostic marker in neonatal hypoxic-ischemic encephalopathy: a study protocol for an individual patient data meta-analysis

**DOI:** 10.1186/2046-4053-2-96

**Published:** 2013-10-25

**Authors:** Pieter LJ Degraeuwe, Gerald J Jaspers, Nicola J Robertson, Alfons GH Kessels

**Affiliations:** 1 Department of Pediatrics, Maastricht University Medical Centre, P. Debyelaan 25, PO Box 5800, 6202AZ Maastricht, The Netherlands; 2 Department of Neonatology, UCL Institute for Women’s Health, 86-96 Chenies Mews, WC1E 6HX London, England; 3 Department of Clinical Epidemiology and Medical Technology Assessment, Maastricht University Medical Centre, PO Box 5800, 6202 AZ Maastricht, The Netherlands

**Keywords:** Hypoxic-ischemic encephalopathy, Infant-newborn, Magnetic resonance spectroscopy, Meta-analysis as topic

## Abstract

**Background:**

The prognostic accuracy of ^1^H (proton) magnetic resonance spectroscopy (MRS) in neonatal hypoxic-ischemic encephalopathy has been assessed by a criticized study-based meta-analysis. An individual patient data meta-analysis may overcome some of the drawbacks encountered in the aggregate data meta-analysis. Moreover, the prognostic marker can be assessed quantitatively and the effect of covariates can be estimated.

**Methods:**

Diagnostic accuracy studies relevant to the study topic were retrieved. The primary authors will be invited to share the raw de-identified study data. These individual patient data will be analyzed using logistic regression analysis. A prediction tool calculating the individualized risk of very adverse outcome will be devised.

**Discussion:**

The proposed individual patient data meta-analysis provides several advantages. Inclusion and exclusion criteria can be applied more uniformly. Furthermore, adjustment is possible for confounding factors and subgroup analyses can be conducted. Our goal is to develop a prediction model for outcome in newborns with hypoxic-ischemic encephalopathy.

## Background

Hypoxic-ischemic encephalopathy (HIE) in the newborn is associated with brain energy metabolism disturbances that can be quantified *in vivo* by ^1^H (proton) magnetic resonance spectroscopy (MRS) [1,2]. A recent study-level meta-analysis demonstrated that deep gray matter lactate/N-acetyl aspartate (Lac/NAA) peak/area ratio has a better prognostic accuracy than conventional and diffusion-weighted MRI for neurodevelopmental outcome after HIE [3].

The suggestion that Lac/NAA might support early clinical decisions was criticized for several reasons [4]. Although based on available studies, the validity of the meta-analysis and the generalizability of the results were questioned [4]. It was suggested that the spectrum of the included patients was too broad, including very mildly (Sarnat 1) and very severely (Sarnat 3) affected patients; unclear selection criteria could undermine generalizability. In addition, some studies were restricted to surviving infants and the timing of the magnetic resonance (MR) studies varied. Death after HIE frequently follows decisions to withdraw life-sustaining care. Hence death as an adverse outcome may cause incorporation bias. It is conceivable that the outcome assessors were not always blinded to the MR results. Precise definitions of adverse outcome were lacking in studies. Concern was also expressed with respect to the *post-hoc* choice of ‘cut-off values’. Wilkinson [4] concluded: ‘It is not possible from published data to assess the usefulness of quantitative markers such as lactate/NAA peak/area ratio for predicting very adverse outcome […] or to look separately at its usefulness for infants with moderate encephalopathy’.

An individual patient data (IPD) meta-analysis, where the raw data from multiple studies are synthesized, may overcome some of the drawbacks encountered in the aggregate data meta-analysis. A prerequisite is that all authors of the original studies are willing to share the individual test results and the patient characteristics to be evaluated. Subgroup analysis based on different HIE Sarnat stage is possible. Infants who died after treatment withdrawal can be excluded from the analysis. An agreed-upon definition of very adverse outcome can be applied uniformly. Finally, logistic regression modeling can be used to derive a prediction tool that calculates the individualized risk of very adverse outcome.

### Objectives

The aims of the planned study are to reassess the prognostic performance of MR biomarkers in neonatal HIE and to determine the effect of other patient variables on the outcome using IPD meta-analysis. Logistic regression modeling will be used to develop a clinical risk prediction rule to assess the individual probability for adverse outcome after HIE.

## Methods

Identification, selection, and appraisal of relevant studies have already been carried out independently, by two reviewers (PLJD, GJJ). Disagreement was resolved through discussion.

### Inclusion criteria for studies

All studies, cohort and case–control studies, evaluating the prognostic accuracy of MRS biomarkers in term and near-term newborns with HIE (Sarnat stages 1, 2 and 3) were considered for review. Case–control studies are prone to spectrum bias [5], and the prevalence affects the predictive value or the post-test probability. Fortunately, in the regression equation:

$$\mathrm{logit}\left(p\right)=\beta_{0}+\beta_{1}.x_{1}+\beta_{2}.x_{2}+\ldots +\beta_{n}.x_{n}$$

only *β*_0_ is dependent on the prevalence. This constant *β*_0_ can easily be readjusted to another (disease or) outcome prevalence.

The following data needed to be available for the study to be included in the IPD meta-analysis:

● MR spectroscopy data for Lac/NAA, Lac/creatine (Cr), Lac/choline (Cho), NAA/Cho, NAA/Cr, or Cho/Cr.

● Reliable, quantitative (numerical) neurodevelopmental outcome data at the age of at least 1 year (and preferably 2 years).

### Search strategy

A systematic search was performed from inception until 14 April 2012 in MEDLINE (Ovid), EMBASE (Ovid), DARE [6], and Medion [7]. No diagnostic search filter [8,9] or language restrictions were used. Details of the search are given in Additional file 1. Any duplicate articles identified were manually deleted. The reference lists of selected studies were checked for further relevant studies.

**Figure 1:**
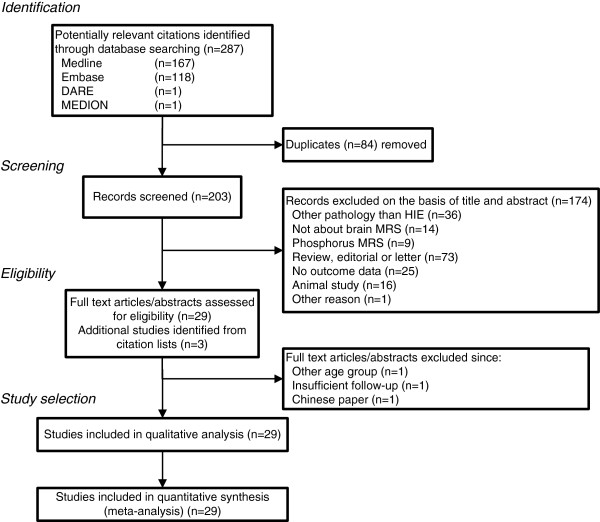
**Flowchart showing search for and selection of papers evaluating prognostic value of **^**1**^**H-MRS in neonatal hypoxic-ischemic encephalopathy.** Final search carried out on 14 April 2012.

After removal of duplicates in our search (see Figure 1), we identified 203 studies of which 174 were excluded on the basis of title or abstract. Another three were excluded after assessment of the full text, and an additional three studies were identified through the reference lists, leaving 29 studies of which the authors will be contacted by email [10-38].

To confirm the thoroughness of our search, the authors will be asked whether they are aware of any untraced but eligible study.

### Quality assessment

**Figure 2:**
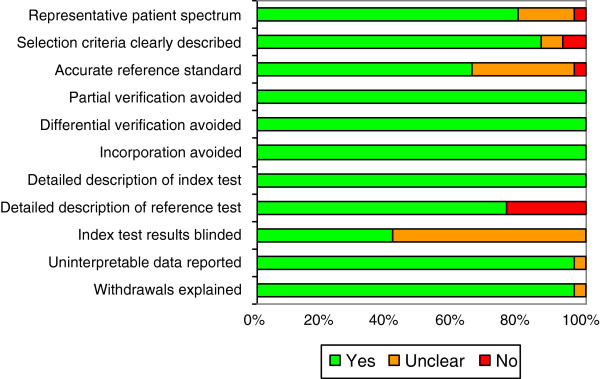
Results of quality assessment using QUADAS tool.

The quality of the 29 selected studies was assessed using the revised QUADAS tool [39,40]. Since MRS is an objective measurement and outcome is always assessed at a later stage, 3 of the 14 items in the QUADAS tool were omitted: time between index and reference test too long, blinded interpretation of the index test, and availability of clinical data. Two reviewers (PLJD, GJJ) independently answered the 11 remaining questions in the affirmative, in the negative or as being unclear. Figure 2 shows the quality of the 29 selected papers.

### Data items to be requested from the authors of the original diagnostic studies

1. Gestational age at birth.

2. Birth weight.

3. Sex.

4. Apgar score at 1, 5, and 10 minutes.

5. Umbilical artery pH and base excess (or first arterial pH and base excess).

6. Sarnat score.

7. Was there an intrapartum sentinel event (for example, uterine rupture, placentalabruption, cord prolapse, and amniotic fluid embolism)?

8. Hypoglycemia: lowest blood (or plasma) glucose in the first 24 hours of life [41].

9. Hyperoxia and hypocarbia: highest arterial pO_2_ and lowest arterial pCO_2_ in the first day of life [42,43].

10. Hypothermia (head or whole body cooling).

11. ^1^H-MRS biomarkers (timing, region of interest (ROI), echo time TE used): peak-area ratios of Lac/NAA, Lac/Cr, Lac/Cho, NAA/Cr, NAA/Cho, Cho/Cr.

12. Outcome (at most recent assessment):

a. Motor function assessed by the Gross Motor Function Classification System (GMFCS) [44]. This five-level classification system describes the gross motor function of children and youths with cerebral palsy on the basis of their self-initiated movement with particular emphasis on sitting, walking, and wheeled mobility. A criteria list for scoring a child in different languages is available [45].

a. Early cognitive function as assessed by Griffiths Mental Developmental Scales (general cognitive quotient), scores on the Bayley II Mental Developmental Index or the Bayley III Cognitive Scale, or similar. For comparison, the raw scores will be converted to Z-scores to account for different standard deviations of the different test result distribution. Children who are too disabled for cognitive testing will be assigned a Z-score of −4. Since early developmental testing is specific but not sensitive for later (school age) impairment [46], cognitive assessment at later age would improve the prognostic accuracy.

a. Visual: bilateral blindness.

a. Auditory: hearing loss requiring bilateral amplification.

13. Was death due to withdrawal of treatment?

The IPD will be delivered by the collaborating researchers using a spreadsheet form for completion in such a way that re-identification is impossible. The data set should not contain personal identifiers such as names, initials, addresses, ZIP code, phone numbers, date of birth or admission, medical record number, social security number, or other unique identifying numbers, characteristics, or codes.

### Definition of adverse outcome

Adverse outcome will be defined as the presence of death, substantial motor dysfunction (severe motor impairment) with a level of III or worse on the GMFCS, bilateral blindness (or only light perception), and/or a developmental quotient of less than 3 standard deviations below the norm [47]. Alternative definitions can be used.

### Data synthesis and (statistical) analysis

The MRS biomarkers of interest are Lac/NAA, Lac/Cr, Lac/Cho, NAA/Cho, NAA/Cr, and Cho/Cr. The contribution to the adverse prognosis of HIE of these biomarkers (as continuous variables) and the clinical characteristics will be explored using stepwise forward binary logistic regression analysis. The logit and logistic command in Stata/SE 10.1 will be used (Stata Corporation, College Station, TX, USA). An entry probability for each variable will be set at 0.05. A clinical prediction rule will be derived from the final regression model. The predictive accuracy of the logistic regression models will be assessed by computing a cross classification table (lstat command) and by ROC curve analysis or c-statistics (roctab and roccomp command in Stata).

Between-study heterogeneity will be assessed by entering the study as a categorical variable in the logistic regression analysis.

The potential for publication bias will be estimated by using a Deeks’ funnel plot. A *P* value <0.1 was considered statistically significant [48].

### Planned sensitivity analyses

The logistic regression analysis will be performed in surviving infants and in survivors plus infants whose decease was not due to a withdrawal decision.

### Ethical considerations

The research will be conducted in accordance with the code of conduct for medical research of the Dutch Federation of Biomedical Scientific Societies [49].

The medical ethics committee at Maastricht University Medical Centre did not make an objection to the proposed IPD meta-analysis, but added the express condition that the patient data will be de-identified.

### Publication policy

The results of the proposed IPD meta-analysis will be published on behalf of all researchers sharing a usable data set. At least one delegate per study will be proposed as co-author to the journals where the meta-analysis will be submitted. A larger study size may be represented by more authors. Alternatively, the paper will be published under a group name, allowing citation of all contributors in PubMed. The manuscript will be circulated to the collaborators for comments, amendments, and approval before being submitted.

The data provided by the authors will be treated confidentially and will not be copied or distributed elsewhere. Furthermore they will not be used for any other publication without the authors’ approval.

### Registration

The protocol has not been registered with PROSPERO (the international prospective register of systematic reviews).

## Discussion

### Strength of the proposed study

Individual participant data meta-analysis has been described as the ‘gold standard’ for prognostic accuracy studies [50]. In the proposed study, we hope to take advantage of several potential advantages of IPD meta-analysis [51,52]. Inclusion and exclusion criteria can be used more uniformly across studies, and overlapping sets of participants can be identified. Adjustments can be made for confounding factors, subgroup analyses can be conducted, and a prognostic model can be generated.

Important but challenging is the fact that follow-up information on neurodevelopmental outcome can be updated and uniformly described. To increase comparability between the studies, we hope the collaborators will be able to classify the motor outcome using the expanded and revised GMFCS [53]. Updated follow-up information on motor and cognitive developmental status will undoubtedly improve the validity of the prognosis.

### Study weaknesses

We are well aware that availability bias is inevitable. No meta-analysis can correct weaknesses in the contributing individual studies, such as diagnostic review bias [54].

Finally, heterogeneity due to different index test methodology (for example, ROI), and especially due to the use of different reference tests is inevitable [55,56], although for the latter problem conversion algorithms exist [57,58]. The modest predictive value of mental development assessment before the 2nd birthday for later cognitive status remains another concern [59].

### Summary

Achieving a more uniformly defined outcome across studies, the possibility of obtaining longer-term outcome data, the exploration of interaction between MRS biomarkers and patient-level characteristics, and the possibility of sub-analyses, justify this proposed IPD meta-analysis.

## Abbreviations

Cho: Choline; Cr: Creatine; GMFCS: Gross motor function classification system; HIE: Hypoxic-ischemic encephalopathy; IPD: Individual patient data; Lac: Lactate; MR: Magnetic resonance; MRI: Magnetic resonance imaging; MRS: Magnetic resonance spectroscopy; NAA: N-acetyl aspartate; ROI: Region of interest; TE: Echo time.

## Competing interests

The authors declare that they have no competing interests.

## Authors’ contributions

PLJD conceived the study, identified, selected, and appraised the studies and drafted the manuscript. GJJ identified, selected, and appraised the studies. NJR contributed to the study design. AGHK gave methodological and statistical advice. All authors contributed to the critical revision of the manuscript. All authors have read and approved the final manuscript.

### Funding

The writing of this study protocol had no explicit funding.

## Supplementary Material

Additional file 1 Search strategies for the identification of studies: electronic searches. Click here for file
