# Supplementary material for: Magnetic resonance spectroscopy as a prognostic marker in neonatal hypoxic-ischemic encephalopathy: a study protocol for an individual patient data meta-analysis
Source: Syst Rev. 2013 Oct 25;2:96. doi: 10.1186/2046-4053-2-96 (PMC4016296; doi:10.1186/2046-4053-2-96)
Supplement: Additional file 1 — Search strategies for the identification of studies: electronic searches. [file 2046-4053-2-96-S1.pdf]

Additional file 1:

Search strategies for the identification of studies - Electronic searches

A. Search History 14-08-2012 - Ovid MEDLINE(R) 1948 to Present

	Searches	Results
1	exp Magnetic Resonance Spectroscopy/	167053
2	"magneti*".af.	484235
3	"resonan*".af.	503816
4	"spectroscop*".af.	271459
5	MRS.af.	11793
6	exp Asphyxia Neonatorum/ or exp Hypoxia-Ischemia, Brain/ or exp Hypoxia, Brain/	15373
7	"hypoxi*".af.	88071
8	"isch*emi*".af.	269710
9	"encephalopat*".af.	36143
10	brain.af.	1019435
11	2 and 3 and 4	138600
12	1 or 5 or 11	179426
13	7 or 8	341285
14	9 or 10	1039486
15	13 and 14	73625
16	6 or 15	79068
17	12 and 16	1778
18	limit 17 to (humans and "newborn infant (birth to 1 month)")	173

B. Search History 14-04-2012 - EMBASE 1974 to 2012 Week 32

	**Searches**	**Results**
1	exp nuclear magnetic resonance spectroscopy/	91095
2	"magneti*".af.	777572
3	"resonan*".af.	787238
4	"spectroscop*".af.	342696
5	MRS.af.	16063
6	exp brain hypoxia/ or exp brain ischemia/ or exp newborn hypoxia/	98356
7	"hypoxi*".af.	133452
8	"isch*emi*".af.	451687
9	"encephalopat*".af.	51865
10	brain.af.	1413371
11	2 and 3 and 4	146444
12	1 or 5 or 11	154954
13	7 or 8	561058
14	9 or 10	1436825
15	13 and 14	128127
16	6 or 15	147044
17	newborn/	471797
18	12 and 16 and 17	245
